# Alterations in subcortical magnetic susceptibility and disease-specific relationship with brain volume in major depressive disorder and schizophrenia

**DOI:** 10.1038/s41398-024-02862-7

**Published:** 2024-03-26

**Authors:** Shuhei Shibukawa, Hirohito Kan, Shiori Honda, Masataka Wada, Ryosuke Tarumi, Sakiko Tsugawa, Yui Tobari, Norihide Maikusa, Masaru Mimura, Hiroyuki Uchida, Yuko Nakamura, Shinichiro Nakajima, Yoshihiro Noda, Shinsuke Koike

**Affiliations:** 1 Center for Evolutionary Cognitive Sciences, Graduate School of Art and Sciences, The University of Tokyo, Tokyo, Japan; 2 Faculty of Health Science, Department of Radiological Technology, Juntendo University, Tokyo, Japan; 3 Department of Radiology, Tokyo Medical University, Tokyo, Japan; 4 Department of Integrated Health Sciences, Nagoya University Graduate School of Medicine, Nagoya, Japan; 5 Department of Neuropsychiatry, Keio University School of Medicine, Tokyo, Japan; 6 University of Tokyo Institute for Diversity and Adaptation of Human Mind, The University of Tokyo, Tokyo, Japan; 7 The International Research Center for Neurointelligence, University of Tokyo Institutes for Advanced Study (UTIAS), Tokyo, Japan

**Keywords:** Neuroscience, Depression, Physiology

## Abstract

Quantitative susceptibility mapping is a magnetic resonance imaging technique that measures brain tissues’ magnetic susceptibility, including iron deposition and myelination. This study examines the relationship between subcortical volume and magnetic susceptibility and determines specific differences in these measures among patients with major depressive disorder (MDD), patients with schizophrenia, and healthy controls (HCs). This was a cross-sectional study. Sex- and age- matched patients with MDD (*n* = 49), patients with schizophrenia (*n* = 24), and HCs (*n* = 50) were included. Magnetic resonance imaging was conducted using quantitative susceptibility mapping and T1-weighted imaging to measure subcortical susceptibility and volume. The acquired brain measurements were compared among groups using analyses of variance and post hoc comparisons. Finally, a general linear model examined the susceptibility–volume relationship. Significant group-level differences were found in the magnetic susceptibility of the nucleus accumbens and amygdala (*p* = 0.045). Post-hoc analyses indicated that the magnetic susceptibility of the nucleus accumbens and amygdala for the MDD group was significantly higher than that for the HC group (*p* = 0.0054, *p* = 0.0065, respectively). However, no significant differences in subcortical volume were found between the groups. The general linear model indicated a significant interaction between group and volume for the nucleus accumbens in MDD group but not schizophrenia or HC groups. This study showed susceptibility alterations in the nucleus accumbens and amygdala in MDD patients. A significant relationship was observed between subcortical susceptibility and volume in the MDD group’s nucleus accumbens, which indicated abnormalities in myelination and the dopaminergic system related to iron deposition.

## Introduction

The cerebral cortex is responsible for many higher functions, including complex cognitive tasks, decision-making, emotional expression, and language [1]. Meanwhile, the subcortical regions process information from the cerebral cortex and are involved in complex activities related to normal behavior and physiological functions, such as decision-making, reward processing, and motor actions [2]. For example, the globus pallidus plays a central role in motor control [3], the amygdala is involved in the processing of emotions and emotional memory [4], and the nucleus accumbens transmits changes in reward stimuli and plays a crucial role in integrating information from short-term memory into behavioral responses [5].

Major depressive disorder (MDD) and schizophrenia are common mental illnesses that have a considerable negative impact on patients’ lives. For these disorders, brain magnetic resonance imaging (MRI) studies indicated common and disease-specific alterations in brain structure and/or function [6–11]. In fact, previous studies reported altered subcortical volumes in various psychiatric disorders, including MDD and schizophrenia [12–17]. Subcortical regions, including the caudate [12, 13], thalamus, and hippocampus [13], are found to be smaller in MDD patients compared to healthy controls (HCs); however, the study conducted using MRI of 8590 samples from the UK Biobank did not observe any statistically significant differences between individuals with depressive symptoms and HCs in any of the subcortical volumes [14]. In contrast, in schizophrenia several consistent changes in subcortical volume have been reported [15, 16]. The Enhancing Neuroimaging Genetics through Mega-Analysis (ENIGMA) Consortium Schizophrenia Working Group conducted a multicenter meta-analysis and found that patients with schizophrenia had smaller volumes in the hippocampus, amygdala, thalamus, and accumbens and larger volumes in the pallidum compared to HCs [17].

Subcortical brain regions are involved in various neural networks and are rich projection sites for neurons related to important neuromodulators such as dopamine, serotonin, and norepinephrine, as well as target sites for psychotropic drugs [18]. Positron emission tomography (PET) imaging provides evidence for the dysregulation of the dopamine system in patients with schizophrenia and loss of monoamine variability in patients with MDD [19], corresponding to the therapeutic targets. Since dopamine synthesis and metabolism depend on brain iron, basal nuclei have high quantities of iron [20]. Iron affects the synthesis and signaling of neurotransmitters such as dopamine, noradrenaline, adrenaline, and 5-hydroxytryptamine, which are involved in emotions, attention, reward, movement, and various other functions. These neurotransmitters are synthesized by a number of iron-dependent enzymes, including phenylalanine hydroxylase, tyrosine hydroxylase, and tryptophan hydroxylase [21]. Quantitative susceptibility mapping (QSM) is an MRI technique that can be used to measure the magnetic susceptibility of different brain tissue types [22, 23]. This technique can be used to identify various contrasts in the brain, including high-contrast paramagnetic substances such as ferritin and iron in the cortex and deep gray matter; hemorrhage; and microbleeds containing deoxyhemoglobin, methemoglobin, and hemosiderin [24]. Further, QSM can be used to identify low-contrast diamagnetic substances, such as myelin in white matter and calcification in the brain [24]. Therefore, QSM can potentially be used to examine the degrees of iron deposition and myelination, which are important considerations in brain evaluation. Additionally, QSM image contrasts, particularly in subcortical structures, may provide a detailed atlas [25–27]. For example, QSM clearly delineates the nucleus accumbens, internal and external globus pallidus, red nucleus, and substantia nigra, for which contrast on T1-weighted images (T1WI) is difficult to obtain [26].

A significant increase in susceptibility values was observed in the bilateral putamen of severely depressed patients compared to mildly and moderately depressed patients or HCs [28]. In addition, there was a significant increase in local magnetic susceptibility in the subcortical structure, including hippocampus, pituitary, and thalamus in the depressed group compared to the HCs [29]. Furthermore, the magnetic susceptibility of the bilateral substantia nigra, left red nucleus and left thalamus was decreased in patients with first-episode schizophrenia compared to HCs [30]. It is assumed that the magnetic susceptibility in each of these subcortical regions is disease-specific. It may be elevated in MDD and decreased in schizophrenia.

In addition, only a few studies on psychiatric disorders examine the relationship between structural subcortical volume changes and magnetic susceptibility; however, research indicates a negative correlation between volume and magnetic susceptibility in the hippocampus in schizophrenia patients [31]. Increased magnetization rates are not solely determined by iron deposition; a reduction in diamagnetic myelin also contributes to this increase. Quantitative MRI evaluations of myelin content have revealed that patients with MDD exhibit lower levels of myelin throughout the brain, particularly in the nucleus accumbens and the lateral prefrontal cortex [32]. The relationship between volume, which evaluates structural changes, and susceptibility, which reflects changes in myelin and iron deposition, may capture their density, and thus could be an important finding. Most QSM studies on psychiatric disorders to date have focused on measuring magnetic susceptibility and have been limited to case-control group comparisons for a psychiatric spectrum. Furthermore, each brain region is measured manually, a method that is not well-suited for large-scale analysis. Therefore, atlas-based and other methods need to be validated. Therefore, to clarify the relationship between volume and magnetic susceptibility by QSM, we conducted an analysis in which the original multi-echo T2*-weighted images (T2*WIs) were co-registered with T1WIs to improve consistency between QSM analysis and volume analysis by T1WI [33].

Based on previous research, it is hypothesized that compared to HCs, patients with MDD exhibit an increase in magnetic susceptibility in the hippocampus, pituitary, thalamus, and putamen, while patients with Schizophrenia show a decrease in magnetic susceptibility in the substantia nigra, red nucleus, and thalamus. Accordingly, we perform a MRI volumetry analysis combined with QSM atlas to examine whether there are specific differences in subcortical brain volumes and magnetic susceptibility among patients with MDD, patients with schizophrenia, and HCs. Finally, we investigated whether there is any relationship between subcortical volume and magnetic susceptibility in individuals with these psychiatric disorders.

## Methods and materials

### Participants

This cross-sectional study examined 49 patients with MDD, 24 patients with schizophrenia, and 50 HCs. Although the three groups were matched for sex and age, they were significantly different in terms of the estimated (premorbid) intelligence quotient (IQ) and handedness (Table 1). The sample size calculation was based on the results of previous QSM cross-sectional studies on MDD group [28]. Using 80% power and a significance level of 0.005, the sample size calculated for this study was a total of 84 individuals. All the patients were recruited from Keio University Hospital, Tokyo, Japan, and subjected to psychiatric diagnoses conducted by experienced psychiatrists in accordance with “Diagnostic and Statistical Manual of Mental Disorders, Fourth Edition, Text Revision” [34]. HCs were recruited through job recruitment boards, Internet advertisements, and announcements in Keio University Hospital and The University of Tokyo. Participants’ exclusion criteria were as follows: any history of neurological disease, illicit drug or alcohol abuse, head trauma with loss of consciousness, or mass abnormalities and/or abnormalities on conventional diagnostic MRI. In addition, HCs were briefly interviewed by certified psychiatrists or psychologists to exclude any previous or present occurrence of psychiatric disorders. For this study, ethical approval was obtained from the Ethics Committee of The University of Tokyo (21-371) and Keio University Hospital (ID: 20170152; UMIN000028863). All participants provided written informed consent before participating in the study.

**Table 1:** Demographic and clinical characteristics of study participants.

Subgroups	HC		MDD		Schizophrenia		Statistics for 3 groups	
	Mean	SD	Mean	SD	Mean	SD	Statistical value	*p*-Value^a^
*n*	50		49		24			
Male/female	25/25		21/28		13/11		χ^2^ = 0.75	0.6
Age (year)	41	10	40	11	42	12	0.96	0.6
Handedness: right/mixed/left	38/9/3		48/0/1		22/0/2			0.001
JART IQ^b^	108	7	112	6	102	11	χ^2^ = 10.7	0.005
(unknown)	(20)		(25)		(2)			
Illness duration (year)	NA		9	7	14	10	2.21	0.14
Chlorpromazine eq. dose (mg)	NA		NA		406.7	376.2		
Imipramine eq. dose (mg)	NA		217.9	29.3	NA			
HAMD 17	NA		15.8	4.8	NA			
PANSS								
Positive symptom	NA		NA		3.5	3.9		
Negative symptom	NA		NA		5.2	7.7		
General psychopathology	NA		NA		26.5	8.0		

*HAMD* Hamilton Depression Rating Scale, *HC* healthy control, *IQ* intelligence quotient, *JART* Japanese Adult Reading Test, *MDD* major depressive disorder, *NA* not applicable, *PANSS* Positive and Negative Syndrome Scale, *SD* standard deviation.

^a^Kruskal–Wallis rank sum test; Pearson’s chi-squared test; Fisher’s exact test.

^b^To estimate (premorbid) IQ, JART25 and JART50 were used in HCs and disease groups, respectively.

### Demographic and clinical variables

For all participants, handedness was assessed using the Rating Scale of Handedness or the Edinburgh Inventory [35] with the following three categories: left-handed individuals, right-handed individuals, and individuals using both hands. For all participants, estimated (premorbid) IQ was assessed using the 25- or 50-item version of the Japanese Adult Reading Test (JART) [36]. In the schizophrenia group, psychiatric symptoms were assessed by trained psychiatrists or psychologists using the Positive and Negative Syndrome Scale (PANSS) [37]. In this group, medication doses were calculated using the chlorpromazine equivalent dose [38]. For patients with MDD, symptom severity was assessed using the 17-item Hamilton Depression Rating Scale (HAMD) [39]. Finally, we assessed medication doses for imipramine as antidepressant equivalent doses.

### Magnetic resonance image acquisition

All participants were imaged on a 3 T scanner (MAGNETOM Prisma; Siemens Healthineers, Erlangen, Germany) using a 32-channel head/neck coil in Komaba campus, The University of Tokyo. For QSM, a three-dimensional (3D) multi-echo GRE sequence was acquired with the following parameters: repetition time/first echo time = 44.0/3.6 ms, echo spacing = 5.91 ms, number of echoes = 8, flip angle = 15°, field of view = 24 cm, 256 × 256 pixel matrix, slice orientation = axial, voxel size = 0.94 × 0.94 × 1.0 mm, and scan time = 5 min. Further, we used 3D fast spoiled-gradient T1WI to measure subcortical volume with the following parameters: repetition time/echo time = 2400/2.22 ms; flip angle = 8°; field of view = 24 cm; 320 × 320 pixel matrix; slice orientation = sagittal, voxel size; 0.8 × 0.8 × 0.8 mm; and scan time = 5 min.

### Image processing

**Fig. 1:**
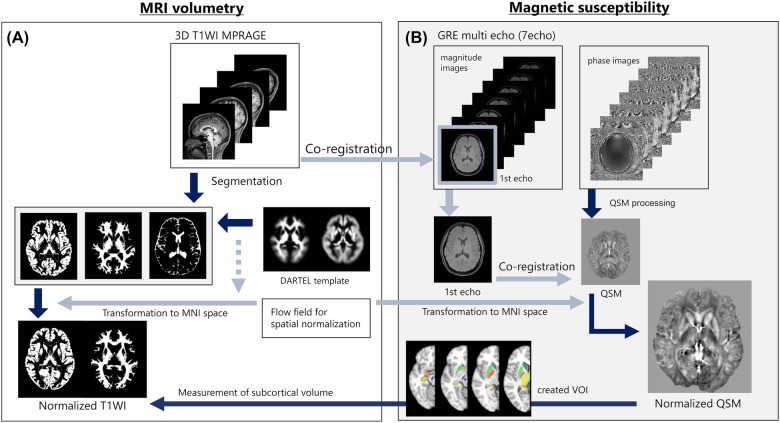
Image-processing steps for quantitative susceptibility mapping (QSM) and T1-weighted imaging (T1WI). **A** MRI volumetry analysis procedure involves the preprocessing of T1-weighted images to generate gray matter, white matter, and cerebrospinal fluid segments. The segmented images are smoothed and registered to a common template space. **B** The procedures of susceptibility estimation and spatial normalization of the map for voxel-based magnetic susceptibility analysis are shown. The susceptibility map is generated from gradient multi-echo magnitude and phase images using a complex fitting algorithm. The resulting susceptibility map is spatially normalized to a standard template space using a nonlinear deformation algorithm to enable group-level statistical analysis.

Figure 1 summarizes the image-processing series. All 3D-T1WIs were segmented into gray matter (GM), white matter (WM), and cerebrospinal fluid (CSF) components using SPM 12. Further, all WM and GM images were used to create the study specific DARTEL template. The T1WIs were converted to Montreal Neurological Imaging (MNI) space maps using the flow field map that was obtained during the study specific DARTEL template creation. Finally, the normalized quantitative images were smoothed using an isotropic 8-mm Gaussian kernel.

QSM was reconstructed from the 3D multi-echo GRE images. Further, the phase images were unwrapped using the Laplacian-based algorithm [40]. Subsequently, to calculate the tissue local field map at each echo, the background field induced by the interaction at the air–tissue interface was removed from the unwrapped phase data by using sophisticated harmonic artifact reduction for phase data with variable kernel sizes ranging from 1 to 29 mm [41, 42]. Each tissue local field map was combined using R2*-based weighted averaging [43]. The QSM image was estimated from the tissue local field map using improved sparse linear equations and the least square method [44, 45]. The zero-reference value was defined as the mean susceptibility value in lateral ventricles [46]. After the reconstruction of QSM images from all subjects, QSM images were warped with standard MNI space maps using the flow field map from T1WI. Finally, QSM was registered to T1WI using the first echo of the GRE in the original image. All calculations were performed in MATLAB 2021a (MathWorks, Natick, Massachusetts, United States).

We created the volume of interest (VOI) manually using general segmentation from the Harvard/Massachusetts General Hospital Center for morphometric analysis as a reference [47] on average MNI QSM maps using ITK-SNAP software version 3.8.0 (Penn Image Computing and Science Laboratory, University of Pennsylvania, United States). Subsequently, we subdivided the 10 subcortical structures red nucleus, substantia nigra, caudate nucleus, putamen, thalamus, hippocampus, nucleus accumbens, amygdala, and globus pallidus externa (GPe) and interna (GPi). GPe and GPi were distinguished using the high contrast of QSM. We evaluated the QSM image using the mean susceptibility value in the spatially normalized susceptibility map without smoothing using the created VOI. Since the subcortex contains small areas, such as the substantia nigra and GPi, two pixels in each slice were eroded inward to reduce the partial volume effect. The volume of each VOI was determined by multiplying the number of voxels within the entire structure with the voxel size in the T1WI image without reducing the size of the created VOI. Each volume and susceptibility value from the left and right hemispheres was averaged for statistical analysis. To confirm the validity of QSM-based volume analysis, we performed a conventional subcortical volume analysis using T1WI and FreeSurfer version 6.0 (Laboratory for Computational Neuroimaging at the Athinoula A. Martinos Center for Biomedical Imaging, Massachusetts, United States) [48] in seven regions (caudate nucleus, putamen, thalamus, hippocampus, nucleus accumbens, amygdala, and pallidum) and confirmed significant relationships between FreeSurfer- and QSM-based subcortical volumes (*r* = 0.24–0.53, *p* < 0.05; Supplementary Material).

### Statistical analyses

All statistical analyses were conducted using R version 4.2.2. We examined differences in demographic characteristics among the groups with MDD patients, schizophrenia patients, and HCs using chi-squared tests for categorical variables and Kruskal–Wallis rank sum test for continuous variables.

We tested the differences in magnetic susceptibility of 10 subcortical regions between the three groups using one-way analysis of variance (ANOVA). Multiple comparisons were corrected using a false discovery rate (FDR). For regions where significant differences were found in ANOVA, post hoc comparisons were conducted using the Tukey honestly significant difference (HSD) test with multiple comparisons corrected using FDR. To test whether the relationship between subcortical volume and magnetic susceptibility differs by group, we applied a general linear model (GLM) with susceptibility as the dependent variable and group (HC, MDD, and schizophrenia), subcortical volume, and their interaction as independent variables. Since volume and magnetic susceptibility have different units, we used standardized variables.

To test the effect of the subject’s handedness, we performed one-way analysis of covariance (ANCOVA) on areas where significant differences were found in ANOVA and post hoc comparisons. Further, to determine the potential effect of IQ, a correlation analysis was performed by group, since a significant amount of data was missing. To clarify the effects of clinical characteristics on magnetic susceptibility, we calculated Pearson correlation coefficients. Specifically, we examined illness duration, symptom severity, and medication dose in the three disease groups for the brain features that indicated significant differences.

## Results

### Differences in brain measures

Data for each group were confirmed for normal distribution using the Shapiro-Wilk test and homogeneity of variances was verified using Levene’s test. Significant group differences were observed in the magnetic susceptibility of the nucleus accumbens (*F* = 5.25, FDR-corrected *p* = 0.045) and amygdala (*F* = 4.90, FDR-corrected *p* = 0.045; Table 2). Post hoc analyses indicated that the magnetic susceptibility of the nucleus accumbens and amygdala for the MDD group was significantly higher than that for the HC group (*t* = 3.16, FDR corrected *p* = 0.0054; *t* = 3.11, FDR corrected *p* = 0.0065, respectively). However, no significant differences were found when comparing the HC and schizophrenia groups or the MDD and schizophrenia groups (*p* > 0.05). No significant differences were also found in any subcortical volume measure (uncorrected *p* > 0.05; Table 3).

**Table 2:** Magnetic susceptibility in subcortical regions.

	Subcortical susceptibility (ppm)^a^			Statistics for 3 groups			Post hoc comparisons
	HC	MDD	Schizophrenia	Statistical value	*p*-value	*q*-value^b^	*p*-value
Red nucleus	0.094 (0.039)	0.099 (0.036)	0.097 (0.035)	0.25	0.80	0.80	–
Substantia nigra	0.11 (0.029)	0.102 (0.029)	0.092 (0.031)	2.72	0.070	0.20	–
Caudate	0.019 (0.010)	0.023 (0.011)	0.020 (0.011)	2.22	0.11	0.22	–
Putamen	0.035 (0.017)	0.038 (0.016)	0.040 (0.019)	1.05	0.40	0.57	–
Thalamus	0.0011 (0.011)	0.0019 (0.011)	0.00010 (0.0088)	0.29	0.70	0.80	–
Hippocampus	–0.012 (0.0079)	–0.0078 (0.0098)	–0.0088 (0.0093)	2.58	0.080	0.20	–
Nucleus accumbens	0.0063 (0.0089)	0.015 (0.015)	0.012 (0.015)	5.25	0.0060	0.045*	0.0054* (MDD vs. HC), 0.139 (Schizophyrenia vs. HC), 0.791 (MDD vs. Schizophrenia)
Amygdala	–0.014 (0.0092)	–0.0073 (0.011)	–0.0097 (0.011)	4.90	0.0090	0.045*	0.0065* (MDD vs. HC), 0.253 (Schizophyrenia vs. HC), 0.624 (MDD vs. Schizophrenia)
Globus pallidus externa	0.11 (0.029)	0.117 (0.024)	0.112 (0.023)	1.01	0.40	0.57	–
Globus pallidus interna	0.11 (0.030)	0.109 (0.025)	0.107 (0.019)	0.18	0.80	0.80	–

*HC* healthy control, *MDD* major depressive disorder.

*<0.05

^a^Mean (standard deviation).

^b^One-way analysis of variance with false discovery rate correction.

**Table 3:** Changes in subcortical volume among groups.

	Subcortical volume (mm^3^)^a^			Statistics for 3 groups		
	HC	MDD	Schizophrenia	Statistical value	*p*-value	*q*-value^b^
Red nucleus	185 (17)	181 (15)	177 (15)	2.02	0.14	0.3
Substantia nigra	480 (45)	471 (38)	461 (39)	1.85	0.20	0.3
Caudate	3044 (311)	3073 (251)	2976 (389)	0.81	0.40	0.6
Putamen	2991 (321)	3032 (268)	2920 (334)	1.10	0.30	0.5
Thalamus	5542 (471)	5476 (450)	5234 (564)	3.38	0.037	0.2
Hippocampus	2682 (217)	2624 (198)	2519 (215)	4.97	0.008	0.085
Nucleus accumbens	316 (33)	310 (27)	304 (38)	1.23	0.30	0.5
Amygdala	1125 (101)	1112 (89)	1075 (105)	2.14	0.12	0.3
Globus pallidus externa	1143 (110)	1149 (88)	1127 (103)	0.39	0.70	0.7
Globus pallidus interna	369 (35)	366 (28)	362 (32)	0.46	0.60	0.7

*HC* healthy control, *MDD* major depressive disorder.

^a^Mean (standard deviation).

^b^One-way analysis of variance with false discovery rate correction.

**Fig. 2:**
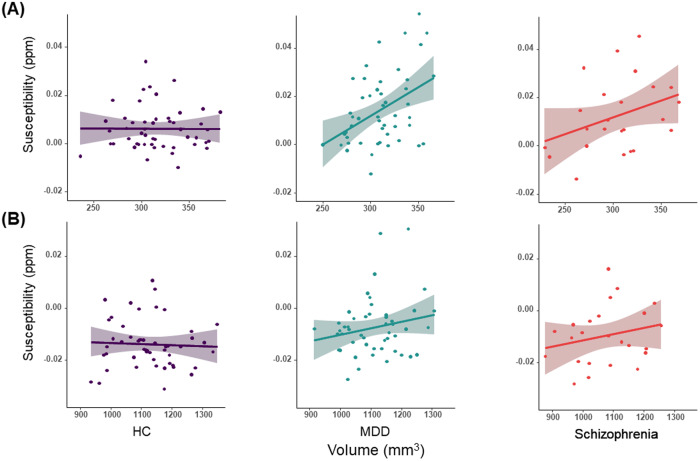
Correlations between volume and magnetic susceptibility in the nucleus accumbens and amygdala. **A** The nucleus accumbens is shown. **B** The amygdala is shown. Each colored area of transparency indicates a 95% confidence interval.

Regarding the magnetic susceptibility of the nucleus accumbens, a GLM showed a significant main effect of group on HC for both MDD (*t* = 3.44, FDR-corrected *p* = 0.0023) and schizophrenia (*t* = 2.33, FDR-corrected *p* = 0.032). Further, a significant group × volume interaction was observed, with larger volume being associated with higher magnetic susceptibility in the MDD group (*B* = 0.58, SE = 0.199, *t* = 2.85, FDR-corrected *p* = 0.015; Fig. 2), but not in the schizophrenia and HC groups (*p* > 0.05). The main effect of volume was not found to be significant (*p* > 0.05). The amygdala indicated a significant main effect of group, with the MDD group being larger than the HC group (*t* = 3.05, FDR-corrected *p* = 0.0085). However, no main effect of volume or group × volume interaction was found in the amygdala.

### Relationship between brain measures and demographic and clinical variables

We conducted ANCOVA with handedness as a covariate and still found psychiatric disorder significant in both the nucleus accumbens (F(2119) = 3.96, *p* = 0.021) and amygdala (F(2119) = 4.00, *p* = 0.020). The correlations between IQ and magnetic susceptibility in the nucleus accumbens and amygdala were not significant (*p* > 0.05) for any groups. Furthermore, we found no significant correlation between the magnetic susceptibility of the nucleus accumbens and clinical variables such as duration of illness, symptom severity, or medication dosage (*r* = 0.16, –0.05, and –0.06 for the corresponding variables; *p* > 0.05) in the MDD group. Similarly, no significant correlations were observed between the susceptibility of the amygdala and the three clinical variables (*r* = 0.27, –0.09, and –0.07 for duration of illness, symptom severity, and medication dosage, respectively; *p* > 0.05).

## Discussion

The present study investigated subcortical magnetic susceptibility and volumetric measures in patients with MDD, schizophrenia, and HCs. Results revealed that, contrary to the hypothesis, patients with MDD had higher magnetic susceptibility in the nucleus accumbens and amygdala compared to HCs, but no significant differences with HCs in any of the subcortical volumetric measures. These findings deviate from the hypothesis that MDD patients would have increased magnetization rates in the hippocampus, pituitary, thalamus, and putamen. However, group × volume interactions indicated the association between larger subcortical volumes and higher magnetic susceptibility of the nucleus accumbens only in the MDD group. This may capture a potential decrease in myelin density, aligning with our predictions.

The study found significant variations in subcortical magnetic susceptibility among the three participant groups. The MDD group had a significantly higher magnetic susceptibility in the nucleus accumbens and amygdala than the HC group, whereas the schizophrenia group showed no significant differences. Contrary to the hypothesis, the MDD group had a significantly higher magnetic susceptibility in the nucleus accumbens and amygdala than the HC group, whereas the schizophrenia group showed no significant differences. Earlier studies on MDD using QSM reported high magnetic susceptibility in the putamen, hippocampus, and thalamus, which is not in agreement with the current results [28]. Further, prior research reported a decrease in magnetic susceptibility in the globus pallidus, left putamen, and left thalamus of first-episode schizophrenia patients [30] and observed high magnetic susceptibility bilaterally in the putamen of chronic schizophrenia patients [31]. The reason for these differences may be that previous study was not an atlas-based analysis using MNI, but a manual ROI analysis [28, 30, 31]. In addition, the current findings are novel since earlier studies did not assess the nucleus accumbens and amygdala. Myelin depletion may contribute to the increased magnetic susceptibility of the nucleus accumbens. Studies using R1, a form of quantitative MRI, have reported myelin reduction in the nucleus accumbens in MDD, which is consistent with the present results [32]. As the nucleus accumbens is speculated to underlie abnormalities in reward processing in MDD, the reduction in myelin may represent reduced neuronal function [49]. Moreover, postmortem studies indicated that patients with MDD recorded a decrease in astrocytes and oligodendrocytes in the amygdala, which probably indicated a decrease in myelin and an increase in magnetic susceptibility in QSM images [50–52]. Myelination depends on activity and is influenced by neural activity; hence, the decrease in myelin may reflect functional abnormalities in the MDD group [53]. Since patients with MDD have reduced activity in the amygdala, changes in magnetic susceptibility in this region are reasonable [54].

Another explanation for the occurrence of significant magnetic susceptibility alterations in the nucleus accumbens and amygdala is increased iron deposition. In patients with MDD, it is believed that there is a state of hypodopaminergic state characterized by reduced production, release, receptor density, and receptor sensitivity of dopamine [55]. Iron deficiency leads to a reduction in the density of dopamine D (2) receptors in the nucleus accumbens, and this density significantly correlates with DA transporter density [56]. Therefore, it is anticipated that iron deficiency may associate the low dopamine system. On the other hand, it has been reported that the high concentration of iron detected in the substantia nigra of Parkinson’s disease patients exceeds the iron buffering capacity of complexes such as neuromelanin, leading to neurotoxicity [57]. Studies using 18F-DOPA PET and QSM have shown that the progression of dopaminergic dysfunction corresponds with iron accumulation in the substantia nigra [58]. Therefore, the impact of iron fluctuations on the dopaminergic system is nonlinear, and further investigation is required to understand the potential influence of iron deposition on the increase in magnetic susceptibility in MDD. Although no significant difference was observed in the present study, it is necessary to increase the number of subjects to verify the relationship between iron deposition and schizophrenia, which is a hyper-dopaminergic disorder. Particularly, a deeper understanding of this issue could be achieved if increases in iron and decreases in myelin could be assessed separately.

The relationship between volume and magnetic susceptibility of the nucleus accumbens showed a significant volume × group interaction. This suggests a link between brain function and brain structure, indicating that magnetic susceptibility plays a role in brain functions such as myelination. However, previous studies in patients with depression and controls have revealed inconsistent relationships between brain structure and functional abnormalities [59]. The assessment of brain function in this study was based on magnetic susceptibility measurements, rather than functional MRI as used in earlier studies. Magnetic susceptibility is known to correlate with quantitative values in PET [58]. Amyloid PET studies of neurodegenerative diseases have shown a relationship with brain volume [60, 61]. Therefore, it is possible that magnetic susceptibility is also associated with brain volume, as is PET.

An increase in magnetic susceptibility would reflect a decrease in myelin, and a correlated increase in volume would consequently observe a decrease in myelin density. Demyelination has been associated with depression-like behavior [62], and the possibility of reduced myelin density in the nucleus accumbens in MDD seems reasonable. Furthermore, the present results demonstrate a main effect of magnetic susceptibility that indirectly reflects brain function abnormality. It is possible that in patients with MDD, minute myelination-related changes occur first, which are captured by the QSM. However, the order in which functional and structural brain abnormalities occur in MDD remains unclear. Overall, the significant relationship between magnetic susceptibility and subcortical volume of the nucleus accumbens in the MDD group alone suggests that abnormalities in myelination and the dopaminergic system related to brain function specific to MDD may contribute in part to changes in magnetic susceptibility and subcortical volume, with magnetic susceptibility as the main effect.

This study has several limitations. First, the sample size was relatively small, which may have reduced the statistical power of the analyses, particularly in the case of schizophrenia. Second, the present study did not examine the potential effects of different classes of medications on brain measures in patients with MDD, although many patients were on medication. Our analysis showed no correlation between medication dosage and magnetic susceptibility measurements; however, future studies on the effects of medications on magnetic susceptibility changes will provide more definitive conclusions. Third, differences in pre-morbid IQ were found among the three groups. However, due to around half of missing data in the MDD group, statistical analysis could not be performed to account for the effect of IQ.

In conclusion, this is the first study to focus on the alterations of magnetic susceptibility using the QSM among MDD, schizophrenia, and HC. The results revealed changes in magnetic susceptibility in the nucleus accumbens and amygdala, particularly in patients with MDD. Moreover, there was a significant relationship between magnetic susceptibility and subcortical volume in the nucleus accumbens only in the MDD group, suggesting that the MDD-specific abnormality of myelination and dopaminergic system may partially contribute to the alteration of magnetic susceptibility as well as subcortical volume. These findings provide new insights into the underlying mechanisms implicated in these disorders and highlight the importance of investigating the relationship between subcortical volume and magnetic susceptibility.

### Supplementary information

Supplementary Figure S1. Correlation scatter plot of a subcortical volume measurement using SPM and FreeSurfer.

## Data Availability

The source code and scripts that support study findings are available from the corresponding author, SK, upon reasonable request.
