# Supplementary material for: Alterations in subcortical magnetic susceptibility and disease-specific relationship with brain volume in major depressive disorder and schizophrenia
Source: Transl Psychiatry. 2024 Mar 26;14:164. doi: 10.1038/s41398-024-02862-7 (PMC10965930; doi:10.1038/s41398-024-02862-7)
Supplement: Supplementary file 1 — Supplementary Figure S1. Correlation scatter plot of a subcortical volume measurement using SPM and FreeSurfer. [file 41398_2024_2862_MOESM1_ESM.docx]

**Subcortical morphometry and magnetic susceptibility analysis in psychiatric disorder**

Supplementary Materials

**Table contents**

[**Supplementary Figures** 2](#_Toc136617720)

[**Supplementary Fig. S1. Correlation scatter plot of the subcortical volume measurement using SPM and FreeSurfer.** 2](#_Toc136617721)

# **Supplementary Figures**

**
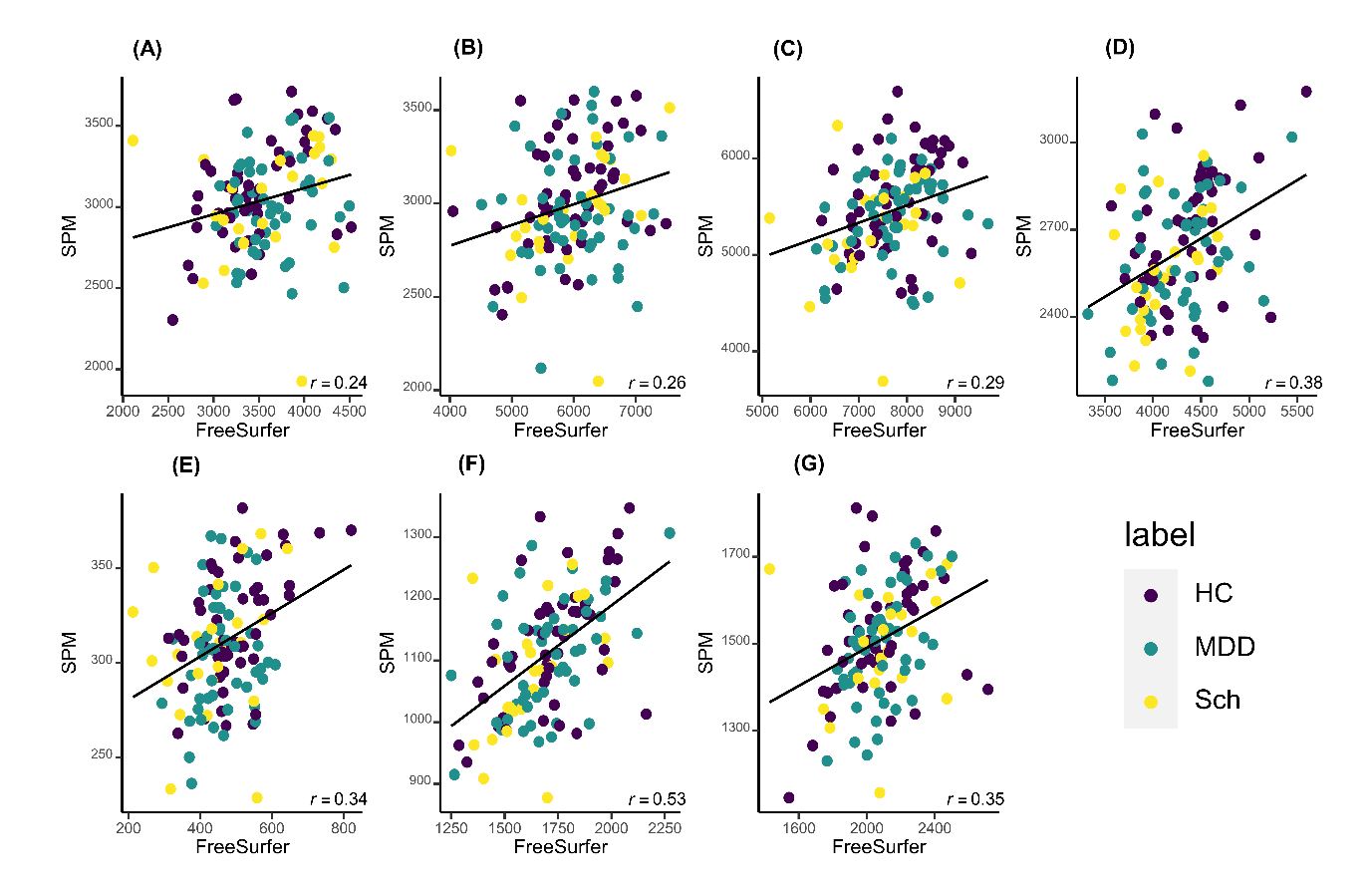
**

## **Supplementary Fig. S1. Correlation scatter plot of the subcortical volume measurement using SPM and FreeSurfer.**

This figure illustrates scatter plots depicting the correlation between the volumetric analyses of the subcortex using SPM in our method and FreeSurfer. The following regions are presented: (A) caudate, (B) putamen, (C) thalamus, (D) hippocampus, (E) nucleus accumbens, (F) amygdala, and (G) globus pallidum.
